# Supplementary material for: Reconciling Mining with the Conservation of Cave Biodiversity: A Quantitative Baseline to Help Establish Conservation Priorities
Source: PLoS One. 2016 Dec 20;11(12):e0168348. doi: 10.1371/journal.pone.0168348 (PMC5173368; doi:10.1371/journal.pone.0168348)
Supplement: S1 Dataset — (ZIP) [file pone.0168348.s002.zip › Taxa/Serra Norte/SN_2013/N4E/Lista N4E-72.pdf]

CAVIDADE N4E-0072

Classe	Ordem	Fam/Outros	Gên/Outros	Espécie	S-U	U-S	Zon.
Annelida	Oligochaeta			jovem		6	P
Arachnida	Acari	(Oribatida)		sp.2	X		P
Arachnida	Acari	(Oribatida)		sp.8		X	P
Arachnida	Acari	(Parasitengonina)		sp.12		X	P
Arachnida	Acari	(Parasitengonina)		sp.3	X		P
Arachnida	Acari	(Parasitengonina)		sp.5		X	P
Arachnida	Acari	Cunaxidae		sp.1		X	P
Arachnida	Acari	Neothyridae	*Diplothyrus*	sp.1	X	X	P
Arachnida	Acari	Prostigmata		sp.49	X		E/P
Arachnida	Acari	Rhagidiidae		sp.2		X	P
Arachnida	Acari	Rhagidiidae		sp.4	X		
Arachnida	Acari	Rhagidiidae		sp.5		X	P
Arachnida	Acari	Trombiculidae		sp.39	X	X	E/P
Arachnida	Acari	Veigaiidae		sp.1	X	X	P
Arachnida	Amblypygi	Charinidae	*Charinus*	sp.1	3	8	P
Arachnida	Amblypygi	Phrynidae	*Heterophrynus*	*longicornis*	1	2	P
Arachnida	Araneae	Corinnidae		jovem	1		P
Arachnida	Araneae	Ctenidae		jovem	2	3	P
Arachnida	Araneae	Drymusidae	*Drymusa*	*spelunca*	X	X	P
Arachnida	Araneae	Gnaphosidae		jovem		X	P
Arachnida	Araneae	Ochyroceratidae	*Ochyrocera*	sp.1	X	X	E/P
Arachnida	Araneae	Ochyroceratidae	*Ochyrocera*	sp.3	X	X	P
Arachnida	Araneae	Ochyroceratidae	*Ochyrocera*	sp.4	X	X	P
Arachnida	Araneae	Ochyroceratidae	*Speocera*	sp.1	X	X	E/P
Arachnida	Araneae	Oonopidae	*Triaeris*	*stenaspis*		X	P
Arachnida	Araneae	Pholcidae	*Mesabolivar*	sp.1	X	X	P
Arachnida	Araneae	Theridiidae	*Theridion*	sp.1	X	X	P
Arachnida	Araneae	Theridiosomatidae	*Plato*	sp.1	X	X	E/P
Arachnida	Opiliones	Escadabiidae		sp.2	X	X	P
Arachnida	Opiliones	Sclerosomatidae	*Prionostema*	sp.1	3		P
Arachnida	Opiliones	Stygnidae	*Protimesius*	*gracilis*	3	1	E/P
Arachnida	Palpigradi	Eukoeneniidae	*Allokoenenia*	sp.1	X	X	P
Arachnida	Pseudoscorpiones	Bochicidae		sp.1	X	X	P
Arachnida	Pseudoscorpiones	Chthoniidae	*Pseudochthonius*	sp.1	X	X	P
Arachnida	Ricinulei	Ricinoididae	*Cryptocellus*	jovem		1	P
Arachnida	Schizomida	Hubbardiidae	aff. *Rowlandius*	sp.1	X	X	P
Chilopoda	Geophilomorpha	Geophilidae		jovem	1		P
Chilopoda	Scolopendromorpha	Cryptopidae	*Cryptops*	jovem		2	P
Chilopoda	Scolopendromorpha	Scolopocryptopidae	*Tidops*	jovem		1	P
Diplopoda	Polydesmida	Aphelidesmidae		sp.1	X		P
Diplopoda	Polydesmida	Chelodesmidae		sp.3	5	1	E/P
Diplopoda	Polydesmida	Cyrtodesmidae		sp.1	X	X	P
Diplopoda	Polydesmida	Fuhrmannodesmidae		sp.1	X	X	E/P
Diplopoda	Polydesmida	Paradoxosomatidae		sp.1		1	P
Diplopoda	Polydesmida	Pyrgodesmidae		sp.1	X	X	E/P
Diplopoda	Spirostreptida	Pseudonannolenidae	*Pseudonannolene*	jovem	1		P
Entognatha	Collembola	Isotomidae		sp.1	X	X	P
Entognatha	Collembola	Paronellidae		sp.1	X	X	P
Entognatha	Collembola	Paronellidae		sp.4		X	P

Entognatha	Collembola	Sminthuroidea		sp.2	X	X	P
Entognatha	Diplura	Campodeidae		sp.1		X	P
Insecta	Blattodea			jovem	2	3	P
Insecta	Coleoptera	Carabidae		sp.3	X	X	P
Insecta	Coleoptera	Chrysomelidae		sp.4	X		P
Insecta	Coleoptera	Elateridae		sp.1		X	P
Insecta	Coleoptera	Ptiliidae		jovem		X	P
Insecta	Coleoptera	Staphylinidae	Pselaphinae	sp.27		X	P
Insecta	Coleoptera	Staphylinidae	Pselaphinae	sp.35		X	P
Insecta	Coleoptera	Staphylinidae	Pselaphinae	sp.36	X		P
Insecta	Coleoptera	Staphylinidae	Pselaphinae	sp.8		X	P
Insecta	Diptera	Chaoboridae		sp.	X		P
Insecta	Diptera	Chironomidae		sp.	X		P
Insecta	Diptera	Phoridae	Phorinae	sp.		X	P
Insecta	Diptera	Psychodidae	Phlebotominae	sp.	X	X	P
Insecta	Diptera	Tipulidae	Tipulinae	sp.	X		P
Insecta	Heteroptera	Dipsocoroidea		sp.		X	P
Insecta	Heteroptera	Hebridae		sp.	X	X	P
Insecta	Heteroptera	Reduviidae		sp.	1		P
Insecta	Heteroptera	Veliidae	*Paravelia*	sp.		X	P
Insecta	Homoptera	Cixiidae		jovem	X	X	P
Insecta	Homoptera	Coccoidea		sp.1	X		P
Insecta	Hymenoptera	Diapriidae		sp.1		X	P
Insecta	Hymenoptera	Formicidae	*Acanthognathus*	sp.1		X	P
Insecta	Hymenoptera	Formicidae	*Camponotus*	sp.1		X	P
Insecta	Hymenoptera	Formicidae	*Camponotus*	sp.2	X		E
Insecta	Hymenoptera	Formicidae	*Eurhopalothrix*	sp.1		X	P
Insecta	Hymenoptera	Formicidae	*Hypoponera*	sp.2		X	P
Insecta	Hymenoptera	Formicidae	*Odontomachus*	sp.1		X	P
Insecta	Hymenoptera	Formicidae	*Pachycondyla*	sp.2		X	P
Insecta	Hymenoptera	Formicidae	*Paratrechina*	sp.1	X	X	P
Insecta	Hymenoptera	Formicidae	*Paratrechina*	sp.2	X		P
Insecta	Hymenoptera	Formicidae	*Solenopsis*	sp.1		X	P
Insecta	Hymenoptera	Formicidae	*Trachymyrmex*	sp.1		X	P
Insecta	Isoptera	Rhinotermitidae	*Heterotermes*	sp.	X		E
Insecta	Isoptera	Termitidae	*Nasutitermes*	sp.	X		P
Insecta	Lepidoptera			jovem		X	E/P
Insecta	Orthoptera	Gryllidae	Eneopterinae	sp.	2		P
Insecta	Orthoptera	Phalangopsidae	*Phalangopsis*	sp.	34	89	E/P
Insecta	Psocoptera	Epipsocidae	*Epipsocus*	sp.2	X		P
Insecta	Psocoptera	Psyllipsocidae	*Psyllipsocus*	sp.1	X		P
Insecta	Thysanura	Nicoletiidae		sp.1	X	X	P
Gastropoda	Pulmonata	Bulimulidae	*Naesiotus*	sp.	X	X	E/P
Gastropoda	Pulmonata	Systrophiidae	*Happia*	sp.	X	X	E
Malacostraca	Isopoda	Armadillidae		sp.1		X	P
Malacostraca	Isopoda	Philosciidae		sp.1	X	X	P
Malacostraca	Isopoda	Platyarthridae		sp.1		X	P
Malacostraca	Isopoda	Scleropactidae	*Circoniscus*	*buckupi*		X	P
Malacostraca	Isopoda	Scleropactidae		sp.1	X	X	P
Symphyla		Scutigerellidae		sp.	X		E/P
Amphibia	Anura	Strabomantidae	*Pristimantis*	cf. *fenestratus*	3		P
Mammalia	Chiroptera	Emballonuridae	*Peropteryx*	*kappleri*	1	3	P

Mammalia	Chiroptera	Furipteridae	*Furipterus*	*horrens*		20	P
Mammalia	Chiroptera	Phyllostomidae	*Carollia*	*perspicillata*	2	10	P
Mammalia	Chiroptera	Phyllostomidae	*Diphylla*	*ecaudata*	3		P
